# Supplementary material for: Causal Relationship between Aging and Anorexia Nervosa: A White-Matter-Microstructure-Mediated Mendelian Randomization Analysis
Source: Biomedicines. 2024 Aug 16;12(8):1874. doi: 10.3390/biomedicines12081874 (PMC11351342; doi:10.3390/biomedicines12081874)
Supplement: Supplementary file 1 [file biomedicines-12-01874-s001.zip › Supplementary Figure/Figure S4/Singe SNP plot of MR analysis for TL to ubm-b-1466.pdf]

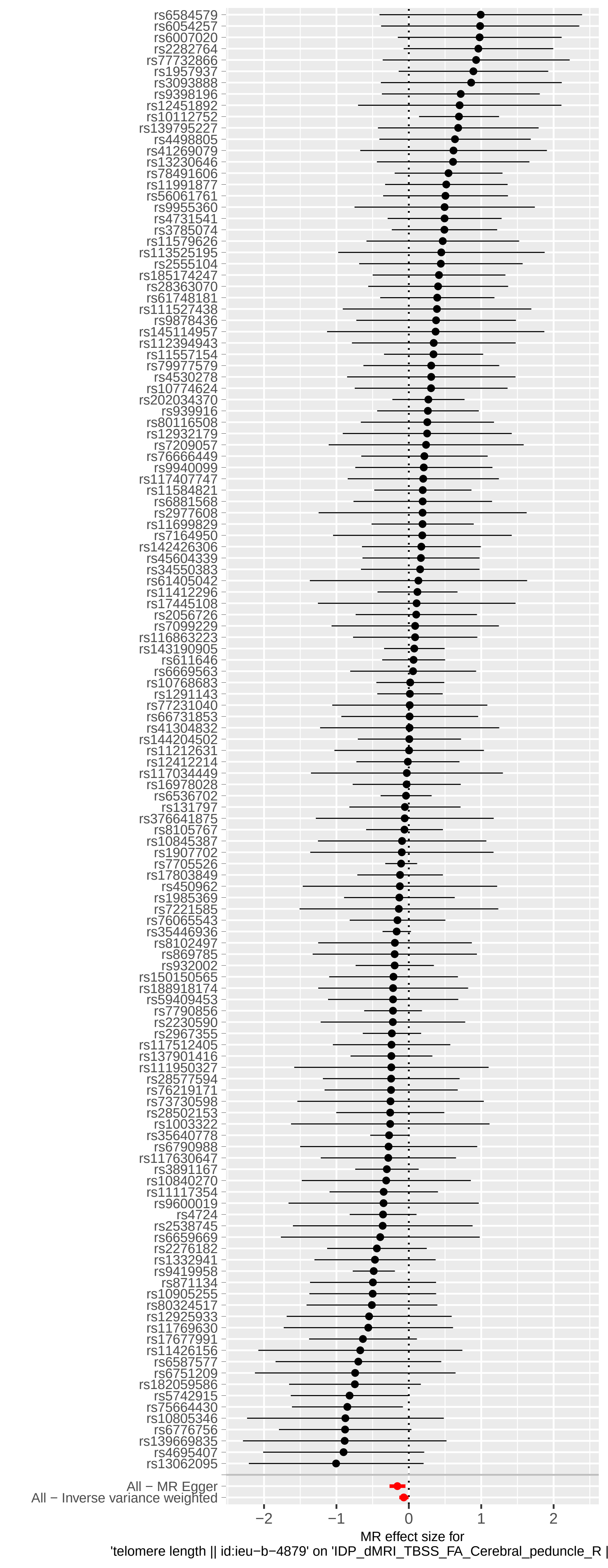
rs6584579
rs6054257
rs6007020
rs2282764
rs77732866
rs1957937
rs3093888
rs9398196
rs12451892
rs10112752
rs139795227
rs4498805
rs41269079
rs13230646
rs78491606
rs11991877
rs56061761
rs9955360
rs4731541
rs3785074
rs11579626
rs113525195
rs2555104
rs185174247
rs28363070
rs61748181
rs111527438
rs9878436
rs145114957
rs112394943
rs11557154
rs79977579
rs4530278
rs10774624
rs202034370
rs939916
rs80116508
rs12932179
rs7209057
rs76666449
rs9940099
rs117407747
rs11584821
rs6881568
rs2977608
rs11699829
rs7164950
rs142426306
rs45604339
rs34550383
rs61405042
rs11412296
rs17445108
rs2056726
rs7099229
rs116863223
rs143190905
rs611646
rs6669563
rs10768683
rs1291143
rs77231040
rs66731853
rs41304832
rs144204502
rs11212631
rs12412214
rs117034449
rs16978028
rs6536702
rs131797
rs376641875
rs8105767
rs10845387
rs1907702
rs7705526
rs17803849
rs450962
rs1985369
rs7221585
rs76065543
rs35446936
rs8102497
rs869785
rs932002
rs150150565
rs188918174
rs59409453
rs7790856
rs2230590
rs2967355
rs117512405
rs137901416
rs111950327
rs28577594
rs76219171
rs73730598
rs28502153
rs1003322
rs35640778
rs6790988
rs117630647
rs3891167
rs10840270
rs11117354
rs9600019
rs4724
rs2538745
rs6659669
rs2276182
rs1332941
rs9419958
rs871134
rs10905255
rs80324517
rs12925933
rs11769630
rs17677991
rs11426156
rs6587577
rs6751209
rs182059586
rs5742915
rs75664430
rs10805346
rs6776756
rs139669835
rs4695407
rs13062095
All − MR Egger
All − Inverse variance weighted
−2
−1
0
1
2
MR effect size for
'telomere length || id:ieu−b−4879' on 'IDP_dMRI_TBSS_FA_Cerebral_peduncle_R |
